# Biocontrol traits of *Bacillus licheniformis* GL174*,* a culturable endophyte of *Vitis vinifera* cv. Glera

**DOI:** 10.1186/s12866-018-1306-5

**Published:** 2018-10-16

**Authors:** Sebastiano Nigris, Enrico Baldan, Alessandra Tondello, Filippo Zanella, Nicola Vitulo, Gabriella Favaro, Valerio Guidolin, Nicola Bordin, Andrea Telatin, Elisabetta Barizza, Stefania Marcato, Michela Zottini, Andrea Squartini, Giorgio Valle, Barbara Baldan

**Affiliations:** 1 Botanical Garden and Department of Biology, University of Padova, Padova, Italy; 2 Department of Biology, University of Padova, Padova, Italy; 3 Department of Biotechnology, University of Verona, Verona, Italy; 4 Department of Chemical Sciences, University of Padova, Padova, Italy; 5 CRIBI Biotechnology Center, Padova, Italy; 6 DAFNAE Department of Agronomy Food Natural Resources Animals and Environment, Legnaro, PD Italy

**Keywords:** Grapevine, Endophytes, Biocontrol bacteria, *Bacillus licheniformis*, Bacterial genome sequencing

## Abstract

**Background:**

*Bacillus licheniformis* GL174 is a culturable endophytic strain isolated from *Vitis vinifera* cultivar Glera, the grapevine mainly cultivated for the Prosecco wine production. This strain was previously demonstrated to possess some specific plant growth promoting traits but its endophytic attitude and its role in biocontrol was only partially explored. In this study, the potential biocontrol action of the strain was investigated in vitro and in vivo and, by genome sequence analyses, putative functions involved in biocontrol and plant-bacteria interaction were assessed.

**Results:**

Firstly, to confirm the endophytic behavior of the strain, its ability to colonize grapevine tissues was demonstrated and its biocontrol properties were analyzed. Antagonism test results showed that the strain could reduce and inhibit the mycelium growth of diverse plant pathogens in vitro and in vivo. The strain was demonstrated to produce different molecules of the lipopeptide class; moreover, its genome was sequenced, and analysis of the sequences revealed the presence of many protein-coding genes involved in the biocontrol process, such as transporters, plant-cell lytic enzymes, siderophores and other secondary metabolites.

**Conclusions:**

This step-by-step analysis shows that *Bacillus licheniformis* GL174 may be a good biocontrol agent candidate, and describes some distinguished traits and possible key elements involved in this process. The use of this strain could potentially help grapevine plants to cope with pathogen attacks and reduce the amount of chemicals used in the vineyard.

**Electronic supplementary material:**

The online version of this article (10.1186/s12866-018-1306-5) contains supplementary material, which is available to authorized users.

## Background

Biological control is an increasingly successful and widespread strategy [1] to decrease plant pathogens and the negative effects of agricultural practices on the environment. It uses beneficial microorganisms, either bacteria or fungi, that can counteract plant pathogens and limit the use of chemicals in agriculture [2]. Among these diverse beneficial microorganisms, bacterial endophytes are powerful tools to protect plants from phytopathogens due to their ability to enter and colonize plants. Endophytes are bacteria that can live inside plant tissues and colonize their hosts without causing any signs of plant disease. They penetrate plants mainly from the soil and roots and spread into leaves, flowers and fruits through the vascular plant system [3, 4]. These bacteria spend part of (facultative endophytes) or all (obligate endophytes) their life-cycle inside plants, exploiting this strategic interaction to their advantage [3, 5]. They may both promote the growth of plants and protect them against harmful bacteria and fungi. Endophytes can enhance plant-growth rate and biomass production largely through phytohormone synthesis, nitrogen fixation, phosphate solubilization and ammonium ion production. They protect hosts as biocontrol agents by interacting directly with pathogens and producing many antimicrobial molecules, and/or by competing for nutrients inside the colonized tissues [3, 6]. Biocontrol bacteria also act indirectly, eliciting Induced Systemic Resistance (ISR) in their plant hosts: a plethora of metabolites produced by endophytes activates plant defense priming responses against pathogens [7].

Many endophyte taxa produce lipopeptides (LPs) - molecules that play a crucial role in biocontrol acting directly as antimicrobial/antifungal compounds and as ISR elicitors in plant hosts. These amphiphilic compounds are formed by a short cyclic oligopeptide linked to a lipid tail [8]. The most widely studied LPs belong to the surfactin, iturin and fengycin families, according to their chemical structure. Surfactins are heptapeptides interlinked with a β-hydroxy fatty acid to form a cyclic lactone ring; due to their strong biosurfactant activity, these molecules can readily associate and anchor themselves to the double layer of phospholipids interfering with membrane integrity. Iturins are heptapeptides bound to a β-amino fatty acid chain 14–17 carbons long. Fengycins are lipodecapeptides with an internal lactone ring in the peptidic moiety and a β-hydroxy fatty acid chain (C14-C18) that may be saturated or unsaturated [9]. These molecules, according to their chemical characteristics, fight bacteria, fungi, mycoplasmas and viruses. Due to their strong surfactant power, LPs enable and favor plant colonization by the producer strain hindering pathogenic tissue infection. Bacteria synthesize these families of lipopeptides in a non-ribosomal way through large enzymatic complexes, namely, lipopeptide synthetases. These mega-enzymes are organized in iterative modules that catalyze reactions for lipopeptide production.

Visualization of bacteria inside plants is always difficult as plant tissues are complex and autofluorescent. Recently, molecular techniques employing fluorescent probes that detect bacteria via hybridization have been used to localize and estimate microorganisms within plant organs [4, 10]. Inoculation with strains tagged with green fluorescent protein (GFP) and glucuronidase gene markers has enabled scientists to observe live bacteria inside tissues, which is particularly useful when following bacterial colonization patterns and estimating endophytic populations [11–13]. GFP-tagged bacteria are handy tools to examine endophyte–plant interactions [14], as GFP does not require any substrate or cofactor to fluoresce. GFP cassettes for chromosomal integration and expression of the reporter gene in many bacterial species have been developed [15–17]. The transformation of bacteria with plasmids harboring integrative cassettes leads to more stable tagged strains, because the chromosomal insertion is less subjected to selective pressure [15]. Recent studies have demonstrated that grapevine cv. chardonnay is efficiently colonized by *Burkholderia phytofirmans* PsJN::*gfp*2x; it colonizes roots, stems and leaves, showing how GFP-tagged strains may be used even to check grapevine colonization [12].

An important characteristic of endophytes is to secrete into the environment lytic enzymes that degrade many biological polymers. Such enzymes, in particular cellulose-lytic enzymes, favor the entrance of endophytes into plant tissues and the formation of stable colonies, giving clear competitive advantage to bacteria with this ability [18]. Before evaluating biocontrol effects, and to develop bacteria inocula for agriculture, it is essential to show if and how a particular strain colonizes inner plant tissues.

In this work, the culturable strain GL174, previously isolated from *Vitis vinifera* cv. Glera [19] was investigated to identify both its endophytic ability and some of its biocontrol traits. GL174 was selected from a collection of putative grapevine endophytes for its plant-growth promotion abilities. Moreover, GL174 can produce ammonia and the plant hormone indole-3-acetic acid, and causes morphological changes to the plant roots when co-cultured with *Arabidopsis thaliana* [20]. The strain was identified as *Bacillus licheniformis* GL174 and its endophytic attitude was validated by plating surface-sterilized inoculated cuttings. Then, using confocal microscopy, we localized the GFP-tagged strain within plant tissues of inoculated grapevine Glera cuttings. Visualization of tagged bacteria inside plant structures allowed us to identify the examined strain as a true endophyte of the plants, and provided a reliable protocol for cutting inoculation for further biocontrol experiments. As the plant growth-promoting (PGP) abilities of strain GL174 had already been demonstrated [20], we focused on its potential biocontrol activity and antifungal properties. We reported both antifungal activity against some grapevine fungal pathogens by an in vitro bioassay and in vivo on grapevine leaves. Furthermore, we reported an effective production of LPs, detected by mass spectrometric analyses and we also sequenced the entire genome. The results of this multidisciplinary approach enabled us to assess the complex pattern of biocontrol traits displayed by *Bacillus licheniformis* GL174*.*

## Methods

### Bacterial strain and growth conditions

The strain GL174 was previously isolated from surface-sterilized tissues of *Vitis vinifera* cv. Glera and identified as *Bacillus licheniformis* [19]. This strain was cultivated routinely in Nutrient Broth (NB) or Nutrient Agar (NA) at 28 °C.

### Grapevine cutting re-inoculation for endophytic proficiency validation

Sterile stem cuttings of grapevine cultivar Glera, approximately 20 mm long, each bearing an axillary bud, were obtained from in vitro plants grown in Murashige and Skoog (MS) solid medium. The GL174 strain was grown overnight in NB medium at 28 °C under shaking. Cells were harvested by centrifugation at 1500×*g* and resuspended in sterile 10 mM MgSO_4_ with a final optical density of 0.1. The lower extremity of each cutting was dipped for 1 min into the bacteria suspension. Negative controls were performed by dipping cuttings into sterile MgSO_4_. Inoculated cuttings were cultivated in MS/2 solid medium, without sugar, at 24 °C, with 25 μmol m − 2 s − 1 light intensity and a photoperiod of 8 h of light and 16 h of dark. Four weeks after cutting inoculation, the presence of GL174 bacteria inside the plants was tested. Sections of plant stems of each cutting were treated to extract endophytes, as previously described. Three different dilutions of the ground plant material were plated onto NA medium and incubated at 28 °C for 48 h [19]. After this preliminary indication, the endophytic colonization of Glera cuttings by GL174 was assessed using a GFP-tagged strain, GL174::*gfp,* and laser scanning confocal microscopy.

### Transformation of *Bacillus licheniformis* GL174

Plasmid pUT*gfp*2X contains a mini-Tn5 transposon delivery system, a P*psbA*-RBS-*gfp*2X cassette: two *gfp* genes, repeated in tandem, plus the additional 35 bp region containing the Ribosome Binding Site, located downstream the constitutive *psbA* promoter [15].

Bacteria were grown in NA plates added with 50 mg/L Kanamycin for 1 week at 28 °C. In order to transform the strain, electroporation was performed following a modified protocol from Xue et al.*,* [21]. A colony was inoculated in 5 mL of NB 0.5 M sorbitol and grown until it reached 0.9 OD_600_. Cells were cooled in ice and centrifuged at 4000 g for 10 min at 4 °C. Bacteria were resuspended in 1 mL and washed 3 times with 500 μL of cold electroporation medium (10% glycerol, 0.5 M sorbitol and 0.5 M mannitol). After washing, cells were resuspended in 60 μL of electroporation medium and mixed with 2 μL of pUT*gfp*2X plasmid (389 ng/μL). Bacteria were incubated 10 min in ice and transferred in pre-chilled electroporation cuvettes. They were electroporated using a Gene-Pulser (Bio–Rad Laboratories, Richmond, CA) set at 2.5 kV, 200 Ω with a resulting time constant of 4.5–5.4 ms. Immediately after electroporation, 1 mL of NB was added to the transformed cells; then bacteria were incubated at 28 °C under shaking for 3 h. After incubation, 100, 200 and 300 μL of bacteria were plated on NA solid medium supplemented with 30 mg/L Kanamycin and incubated at 28 °C. The resulting colonies were analyzed under a fluorescence stereomicroscope (excitation at 488 nm) to check bacteria fluorescence.

### Inoculation of Glera cuttings with *Bacillus licheniformis* GL174::*gfp2x*

The ability of these strains to colonize and survive within Glera tissues was investigated inoculating cuttings and analyzing the plants by Laser Scanning Confocal Microscopy (LSCM). We used apical cuttings with 2 leaves obtained from 1 month-old plants grown in vitro. One fluorescent colony was inoculated in NB with 30 μg/μL of kanamycin and grown overnight. Bacteria was centrifuged and resuspended in 5 mL of MgSO_4_ 10 mM. The optical density of the suspension was measured and bacteria were diluted with the same buffer to a cell density of 10^6^ cells/mL. One drop (5 μL) of the suspension was placed on the surface of the solid medium MS half-concentrated [22] contained in a vented Magenta box. In correspondence of the drop, one apical cutting was planted in the medium. As negative control, 3 cuttings were inoculated only with MgSO_4_ buffer. In order to check colonization, we inoculated 6 plants for each strain: 3 cuttings were sampled 30 days after inoculation. Stem samples were 3/4 cm long, inclusive from the inoculation point and the first node. One control plant was also analyzed to control the absence of any fluorescence bacteria inside the tissues.

### Laser scanning confocal microscopy of inoculated Glera tissues

Stem explants were first surface-sterilized for 2 min with sodium hypochlorite 1%, rinsed with 70% ethanol and then washed 3 times for 10 min with sterile deionized water. Stems and roots were sliced longitudinally with a blade, and leaf fragments were observed directly. Plant explants were mounted on a slide with a solution of 50% glycerol and covered with a coverslip. Confocal laser scanning microscopy was performed with a Leica SP5 system using an excitation laser of 488 nm (Argon laser) and collecting the emission band of 515–560 nm for GFP fluorescence and of 695–765 nm for chlorophyll fluorescence.

### In vitro antifungal effects of endophytic bacteria

The evaluation of in vitro antifungal effects was performed testing strain GL174 against some grapevine-pathogenic fungi like *Phaeoacremonium aleophilum*, *Phaeomoniella spp*, *Botryosphaeria spp*, *Botrytis cinerea* and more generic plant pathogens *Sclerotinia sclerotiorum* and *Phytophthora infestans*. The endophyte was streaked horizontally in the middle of a Petri dish with PCA medium; plates were incubated at 28 °C for 48 h to obtain bacterial growth. After bacterial growth, two inocula of fungal mycelium were placed on the same plate, one on the right and one on the left of the endophyte. Plates with both bacteria and fungi were incubated for one week at 28 °C. High-resolution pictures (600 dpi) of the plates were obtained and the antifungal effect of the bacterium was evaluated comparing the inhibition of mycelium expansion in the presence of the endophyte strain, and measuring the mycelium radius in the direction of the bacterium using photo-editing software. For each plate we calculated the average radius of the mycelia using the following equation: Rm = ((R1-Rin) + (R2-Rin))/2, where Rin is the fungal inoculum radius. An inhibition index was calculated as percentage of reduction of fungal growth comparing Rm and the mycelium radius of control plates containing fungi without bacteria.

### In vivo antagonism assay

The biocontrol activity against *Botrytis cinerea* was tested using two sets of plants: the first set was represented by 60 day-old plants in soil pots and the second one by 60 day-old plants inoculated with GL174 when propagated as cuttings (see above the described inoculation protocol); to mime the endophyte colonization and check their direct effect, the abaxial sides of leaves were infiltrated with a 10^3^ cells/mL bacteria suspension (in 10 mM MgSO_4_ buffer) by means of a syringe without needle; as negative control some leaves were injected with sterile MgSO_4_ buffer. On these two groups of plants, the antagonism tests were performed on detached leaves [23] and on leaves *in planta*.

In the first test, detached fully expanded leaves from plants, both those infiltrated with GL174 and those from previously GL174-inoculated plants, were placed on wet paper and were challenged by placing a mycelial plug (diameter 10 mm) of *Botrytis cinerea*, collected from a 7-day-old PDA plate, on the middle of the leaves. The trays with the challenged leaves were covered to keep a high relative humidity for the fungus development.

In the second test, leaves of the plants, both those infiltrated with GL174 and those from previously GL174-inoculated plants, were challenged *in planta* with mycelial plugs as described above. Plants were kept in plastic bags in a growth chamber.

Non-treated leaves were included in all the experimental conditions: not infiltrated leaves and leaves from not inoculated plants were challenged with the fungus. Negative controls were also set providing sterile medium plugs to the leaves to check any detrimental effect of the inoculation method independently from the fungus.

The effects of the fungus infection on every set of treated leaves were evaluated after 1 week of infection collecting pictures of the leaves and measuring the surface of the brown lesions by means of the software ImageJ. The mean of the values recorded on bacterized leaves was compared with the mean of the surface values obtained by non-treated leaves. Five plants were used in each treatment: for each treatment three/four leaves were used and data are expressed as damaged area (cm^2^) and asterisks indicate statistically significant differences among treatments (T student test; *p* = 0.05).

### Liquid chromatographic-mass spectrometric analysis

Strain GL174 was analyzed in liquid culture for production of lipopeptides. To obtain the crude extract of LPs, bacteria were inoculated in 500 mL of NB, and grown for 96 h at 28 °C under shaking. Bacteria were removed by centrifugation at 4000 g for 20 min. The supernatant was acidified to pH 2 with 6 N HCl. A white precipitate was obtained and collected by centrifugation at 8000 g. The supernatant was discarded and the solid precipitate extracted with 10 mL of methanol using the volume ratio solid-solvent 1:2. Suspensions were stored at 4 °C for 1 h and the clear methanol phase was transferred into a vial for the electrospray ionization mass spectrometry (ESI-MS) analysis [24, 25].

### Genome sequencing and sequence analysis

Genomic DNA of *Bacillus licheniformis* GL174 was extracted using UltraClean® Microbial DNA Isolation Kit (MoBio, Solana Beach CA, USA) from 5 mL of an overnight culture. Afterwards, Genomic DNA was fragmented and sequenced using ION Proton (Life technologies©) sequencing technology. Genome assembly was performed with the Newbler program.

Sequencing data, assembly and gene prediction were submitted to a public database and are available at BioProject database (http://www.ncbi.nlm.nih.gov/bioproject/) with accession number PRJNA274883. The gene annotation process was performed using the annotation pipeline implemented in the BASys bacterial annotation system (https://www.basys.ca/) so that all the coding sequences were assigned to a COG (Cluster of Orthologs) functional class. In addition, the identified coding sequences were compared with the InterPro database (https://www.ebi.ac.uk/interpro/) for double annotation of the protein functions. Among all the identified protein functions, we isolated the sequences related to chemotaxis and motility, plant wall degrading enzymes and plant colonization, iron nutrition and metabolism, phosphate nutrition and metabolism, nitrogen uptake and metabolism, lipopeptides and other secondary metabolites biosynthesis and oxidative stress response.

## Results

### Strain GL174 can colonize Glera cuttings grown in gnotobiotic conditions

Grapevine Glera cuttings from sterile micro-propagated plants were used to check how *Bacillus licheniformis* GL174 strain colonizes and spreads inside plant tissues. After one month, no bacteria were isolated from control plants, confirming the absence of culturable endophytes in non-inoculated cuttings. We re-isolated the same strain from stems of the in vitro inoculated cuttings (5.66 ± 0.21 log_10_ CFU/g FW) and obtained a preliminary indication that the Glera plant inner tissues of the stem were colonized, and that GL174 is a true endophyte of Glera grapevine.

Analysis of kanamycin resistance demonstrated that *Bacillus licheniformis* GL174 is not resistant to kanamycin: no colonies were detected on NA supplemented with 50 mg/L kanamycin. This result prompted us to use a plasmid bearing a Kanamycin resistance cassette as selective marker. Transformation of *Bacillus licheniformis* GL174 with pUT*gfp*2x provided a fluorescent strain, comparable with the wild type for both growth rate and colony morphology. The GFP-tagged strain was used to follow bacteria inside *Vitis vinifera* Glera and verify that the strain could recolonize plant tissues and thrive within them.

**Fig. 1:**
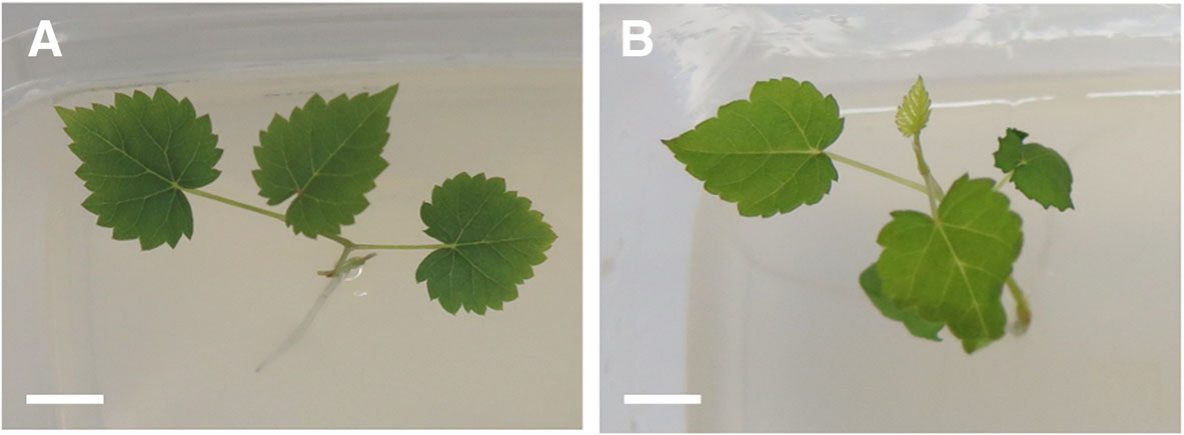
Glera cutting 30 days after inoculation with GL174 (**a**) and control cutting (**b**). Bars: 2 cm

**Fig. 2:**
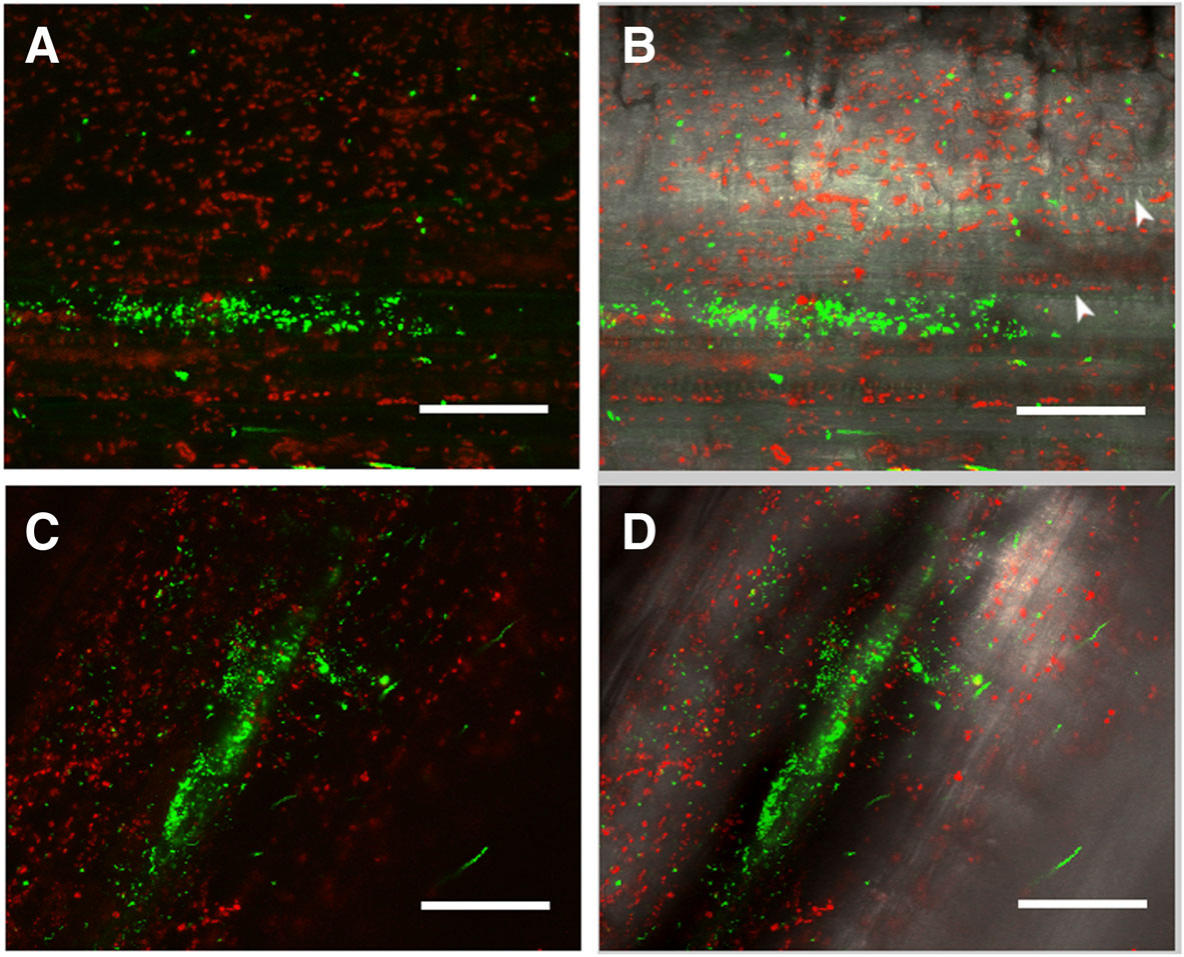
LSCM of stem sections of Glera cuttings inoculated with *Bacillus licheniformis* GL174::*gfp2x*. Overlay of GFP signal (green) and chlorophyll (red) (**a**, **c**) and overlay of fluorescence and bright field (**b**, **d**). Stems samples (**a**, **b**, **c**, **d**) were observed 30 days after inoculation. Arrowheads indicate xylem vessels. Bars: 50 μm

After 30 days, cuttings inoculated with the strain appeared healthy and without any signs of disease-related infection (Fig. 1a and b). We examined longitudinal stem sections (taken at 4 cm from the inoculation point) of three plants and detected bacteria by harvesting their fluorescence inside the stem, mainly located within xylem vessels (Fig. 2a-d).

### *Bacillus licheniformis* GL174 inhibits mycelium growth of some grapevine pathogenic fungi

**Fig. 3:**
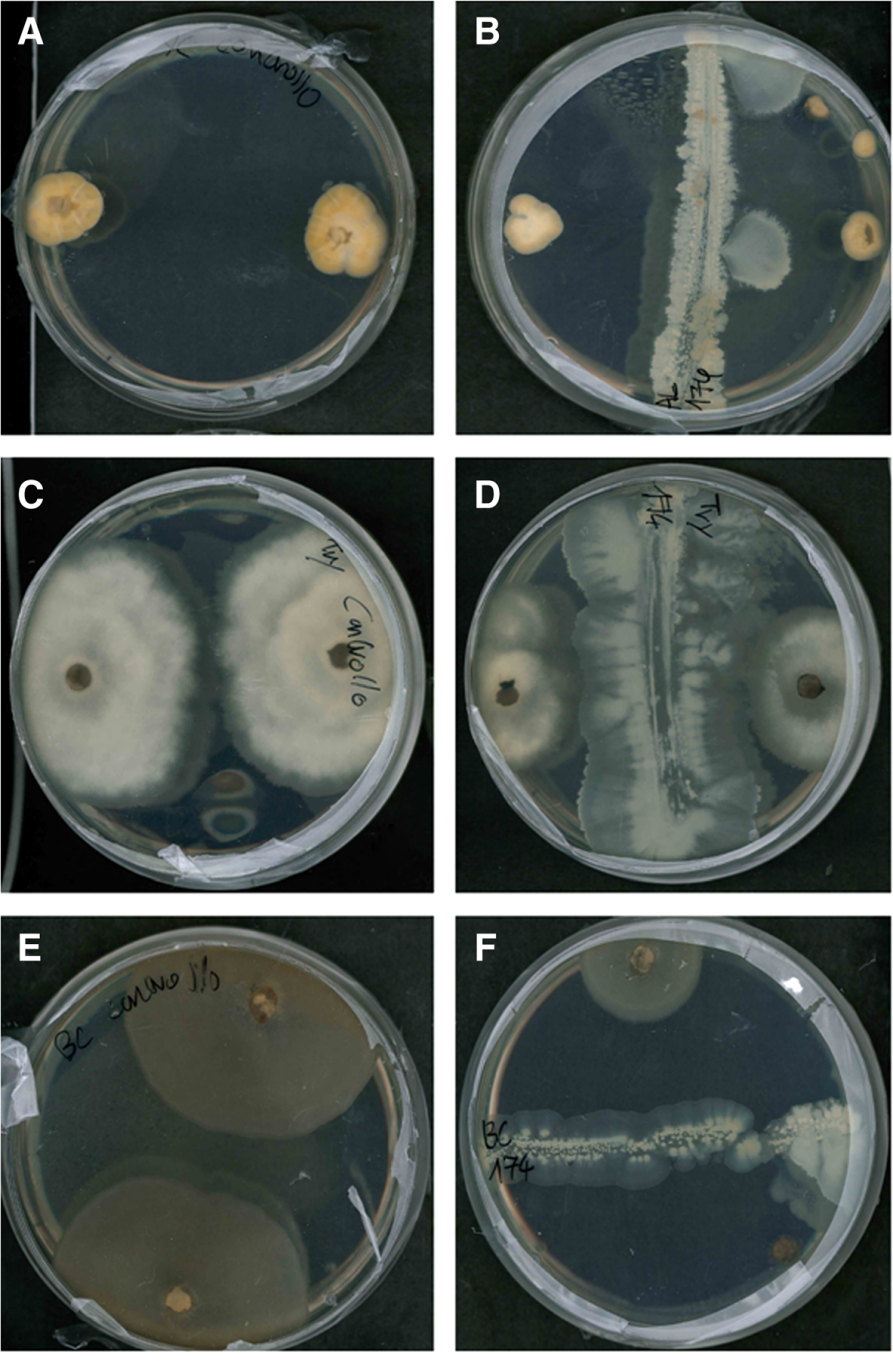
In vitro effect of *Bacillus licheniformis* GL174 on the plant pathogens *Phaeoacremonium aleophilum* (**a**, **b**)*, Botryosphaeria spp* (**c**, **d**)*, Botrytis cinerea* (**e**, **f**). Reduction of mycelium growth due to bacterium action (**b**, **d**, **f**) compared with negative controls without bacteria (**a**, **c**, **e**)

*Bacillus licheniformis* GL174 was challenged to determine in vitro antifungal effects against some pathogenic grapevine fungi: *Phaeoacremonium aleophilum* (Fig. 3a, b)*, Botryosphaeria spp* (Fig. 3c, d)*, Botrytis cinerea* (Fig. 3e, f)*,* and against two more generic plant pathogens: *Phytophtora infestans* (see Additional file 1A, B)*,* and *Sclerotinia sclerotiorum* (see Additional file 1C, D). The antagonistic effect of the strain was quantified by measuring the mycelium radius. Except for *Phytophtora infestans,* all pathogen growth was reduced in the presence of the endophyte; mycelium expansion was inhibited by over 60% compared with the negative controls in which mycelia grew without any antagonist bacteria (Table 1).

**Table 1:** Inhibition Index ± %SE of strain GL174 against some plant pathogens

	*Botrytis cinerea*	*Phaeoacremonium aleophilum*	*Botryosphaeria spp.*	*Phytophthora infestans*	*Sclerotinia sclerotiorum*
Inhibition Index (%)	84.7 ± 11	86 ± 7	63 ± 2	16.3 ± 3	84.4 ± 8

### *Bacillus licheniformis* GL174 reduces *Botrytis cinerea* mycelium growth on grapevine leaves

**Fig. 4:**
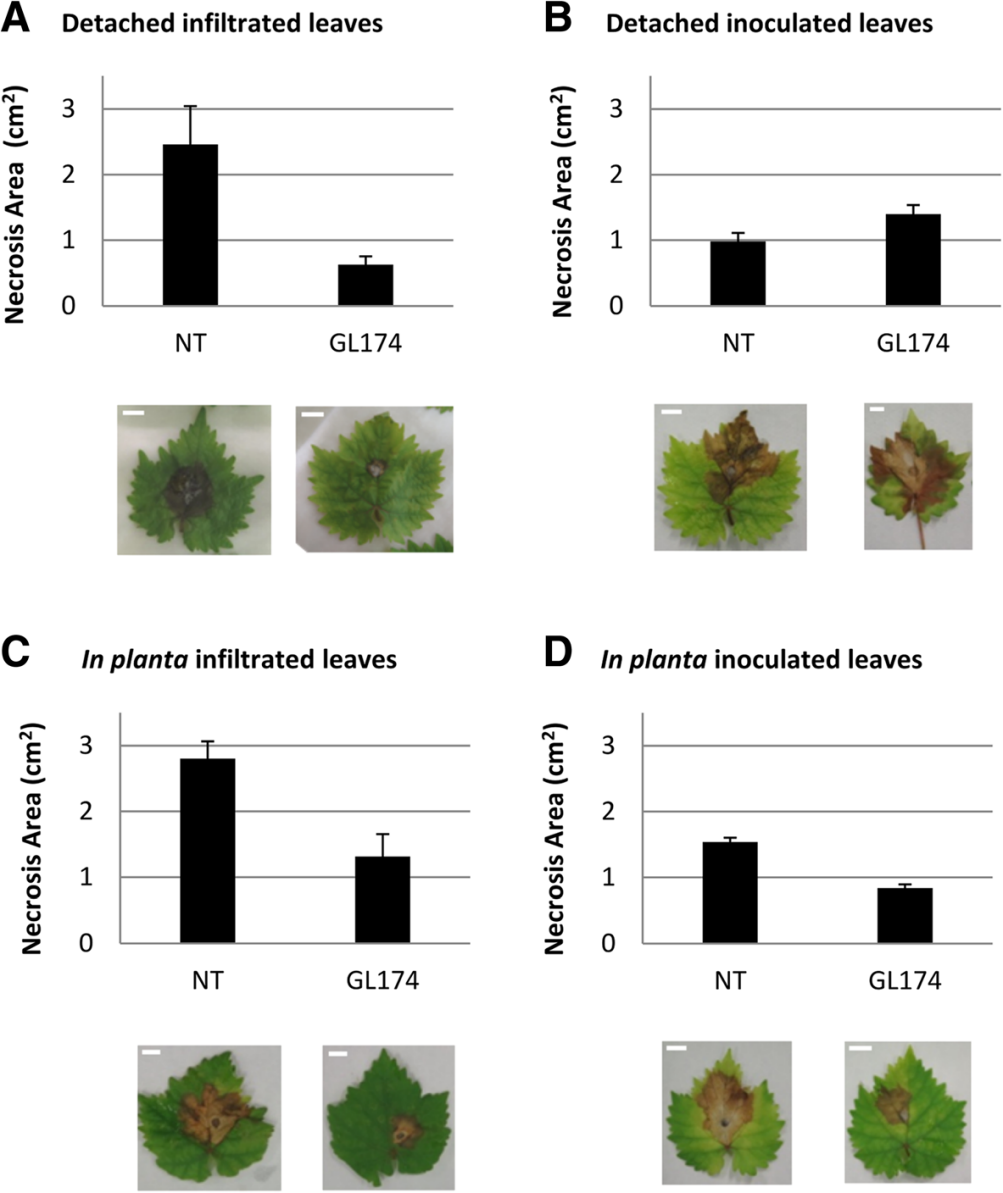
In vivo effect of *Bacillus licheniformis* GL174 presence on grapevine Glera resistance against *Botrytis cinerea*. Symptom severity was determined on detached leaves (**a**, **b**) and on *in planta* leaves (**c**, **d**) in absence (NT) or in presence of bacteria (GL174). Except for (**b**) the plots report statistically significant differences between the treatments (t-student test, *P* < 0.05). Data are reported ± SEM. Example of leaves showing fungal lesions are reported for every set of samples. Bars: 2 cm

The antagonism test on detached and *in planta* grapevine leaves revealed the biocontrol attitude of the examined strain. Detached leaves, infiltrated with strain GL174, showed a significant reduction of the necrotic pathogen-induced area (Fig. 4a) whereas the fungus effect on leaves from two month-old GL174 inoculated plants was comparable with that observed on not bacterized plants (Fig. 4b). *In planta* both GL174 infiltrated leaves and leaves from two month-old GL174 inoculated plants displayed a sharp reduction of area with symptoms compared to non treated plants (Fig. 4c and d).

### Mass spectrometry analysis of GL174 culture supernatant reveals the production of LPs

Considering the results obtained from in vitro and in vivo antagonism assays and from genome analysis indications, the production of LPs in GL174 culture medium was investigated by mass spectrometry analysis. The Ultra-High Performance Liquid Chromatography (U-HPLC) coupled to mass spectrometric detector revealed that *Bacillus licheniformis* can produce several molecules belonging to the surfactin and lichenysin families. Table 2 shows a list of these interesting molecules produced by strain GL174 that were further examined by fragmentation and mass spectrometry analysis, and issued characteristic and accurate *m/z* values of precursors and more abundant fragments by Favaro et al. [26].

**Table 2:** Lipopeptides produced by *Bacillus licheniformis* GL174, determined by LC-MS/MS. Nd: not determined

Lipopeptide	Aminoacidic sequence
Linear surfactin	nd
Cyclic surfactin	Glu1Leu2Leu3Val4mAsp5Leu6Leu7
	Glu1Leu2Leu3Val4Asp5Leu6Leu7
	Glu1Leu2Leu3Val4Asp5Leu6Val7
	Glu1Leu2Leu3Val4mAsp5Leu6Leu7
Linear lichenysin	nd
Cyclic lichenysin	Gln1Leu2Leu3Val4mAsp5Leu6Ile7
	Gln1Leu2Leu3Val4mAsp5Leu6Val7
	Gln1Leu2Leu3Val4Asp5Leu6Val7
	Gln1Leu2Leu3Val4AspLeu6Ile7
	Gln1Leu2Leu3Val4mAsp5Leu6Ile7

### Genome sequencing uncovers the genome structure and the presence of several genes involved in biocontrol and plant-bacteria interaction

The genome of *Bacillus licheniformis* GL174 was sequenced using the IonProton platform, which produced a total of 18,010,684 reads. After filtering the sequences with a quality cutoff, the number of available reads dropped by 13,446,116, for a total of 2,208,443,477 sequenced bases.

Genome assembly was performed with the Newbler program, which yielded 441 contigs. Contigs whose length was lower than 200 bases were filtered, leaving a final dataset of 128 contigs that account for a genome length of 4,208,275 bases with a N50 of 77,942 bp and with an average GC content of 46.9%.

The gene annotation process identified 3902 putative coding sequences (CDS) and 411 pseudogenes, representing a coding density of 87%. Automatic gene annotation allowed us to assign a putative biological function to 2936 genes (75%), while 966 genes (25%) were annotated as “hypothetical protein”. However, we discovered that almost all the CDSs with unassigned function (963) found a homologous gene in the non-redundant database. Using the annotation pipeline implemented in the BASys bacterial annotation system (https://www.basys.ca/), we assigned a COG functional class to 87% of the predicted genes. Amino acid metabolisms and transport (E), carbohydrate metabolism and transport (G) and transcription (K) were among the most abundant classes. All the COG categories found in GL174 were compared with the COG annotation of a reference genome of *Bacillus licheniformis* DSM13 (GCA_000008425.1).

In line with the COG annotation and InterPro (https://www.ebi.ac.uk/interpro/) comparison among the predicted genes, many were involved in biocontrol and plant bacteria interaction (Table 3). The endophytic lifestyle of the strain is apparently helped by a set of gene-coding proteins associated with motility, chemotaxis and plant invasion. The strain has the genes encoding the machinery for flagella biosynthesis and chemotaxis. The genome annotation revealed genes *cheA, cheV, cheY*, che*W* and *motA*. In accordance with previous colonization analyses, genome examination showed several different gene codes for putative lytic enzymes favoring movement through the plant cell wall. COG annotation revealed that many putative cell-wall-degrading enzymes are found within the GL174 genome, such as cellulases, endoglucanases, glucosidase and β-xylosidases. In addition to the last group of cell-wall-degrading enzymes, GL174 has many ABC-type transport system components (periplasmic components and permeases) for the movement of xylose and for its use as carbon source. The strain GL174 genome was found to contain 196 ABC-like transport system-related proteins, as well as 8 putative major facilitator transporters (MFT) and 38 putative phosphotransferase system family (PTS) genes. Among these groups of transport proteins, there were several transporters for phosphate, iron and nitrite/nitrate. We detected 16 gene codes for proteins related to siderophore biosynthesis and iron transport systems. In addition to these transport proteins, the strain also contains a set of putative proteins involved in phosphate metabolism, like alkaline phosphatase, inorganic pyrophosphatase/exopolyphosphatase, 3-phytase and some predicted phosphatases and pyrophosphatases. Gene annotation focused on nitrogen assimilation and metabolism demonstrated that the strain has genes encoding transporters for nitrate, nitrite and ammonium; it also contains nitrite and nitrate reductase that are involved in the nitrogen transformation processes. As potential biocontrol strain, we focused on the genes involved in the production of antimicrobial molecules and those which elicit induced resistance responses in the plant. COG analysis and UNiprot comparison revealed the three sub-units needed for biosynthesis of lipopeptides of the surfactin family, as well as other genes involved in non-ribosomal peptide synthesis. In addition to the putative genes involved in the biosynthesis of non-ribosomal peptides, other gene-encoding enzymes for antibiotic biosynthesis and transport were found. In fact, different sequences have been annotated as enzymes involved in the biosynthesis and transport of lichenicidin, a molecule belonging to the bacteriocin lantibiotics. Inside the genome of the *B. licheniformis* strain we also found some genes associated with the production of spermidine. Furthermore, the genome investigation has provided the sequences of putative genes involved in the biosynthesis of acetoin: the enzyme acetolactate synthase for the production of the acetoin precursor and the enzyme acetoin dehydrogenase that converts acetolactate into acetoin. Moreover, the enzyme responsible of the conversion of acetoin into 2,3-butanedione (acetoine reductase) was found by the UNiprot comparison. The genome analysis has also revealed the presence of two different sequences annotated as putative chitinases, enzymes able to degrade the fungal cell walls. Taking into account the ability of the bacterium to overcome plant response and colonize its tissues, we looked for gene-encoding enzymes for oxidative stress tolerance. We found five different coding sequences annotated as catalase and one sequence annotated as glutathione peroxidase.

**Table 3:** Genes involved in the plant-bacteria relationship found in the *Bacillus licheniformis* GL174 genome

Category	Protein name	Sequence ID	COG definition
Chemotaxis and motility	Probable methyl-accepting chemotaxis protein BT9727_0469	TY90_12985 [C]	COG0840 Methyl-accepting chemotaxis protein
	Chemotaxis protein CheY	TY90_15200	COG0784 FOG: CheY-like receiver
	Flagellar motor switch phosphatase FliY	TY90_15195	COG1776 Chemotaxis protein CheC, inhibitor of MCP methylation
	Motility protein B	TY90_17335	COG1360 Flagellar motor protein
	Methyl-accepting chemotaxis protein tlpC	TY90_17020	COG0840 Methyl-accepting chemotaxis protein
	Chemotaxis protein CheA	TY90_17615	COG0643 Chemotaxis protein histidine kinase and related kinases
	Methyl-accepting chemotaxis protein mcpA	TY90_06200	COG0840 Methyl-accepting chemotaxis protein
	Flagellar motor switch protein FliM	TY90_15190	COG1868 Flagellar motor switch protein
	Probable methyl-accepting chemotaxis protein BT9727_0469	TY90_21790	COG5278 Predicted periplasmic ligand-binding sensor domain
	Motility protein A	TY90_17330	COG1291 Flagellar motor component
	Swarming motility protein swrAA	TY90_02805	na
	Methyl-accepting chemotaxis protein mcpC	TY90_18510	COG0840 Methyl-accepting chemotaxis protein
	Swarming motility protein swrB	TY90_17590	na
	Methyl-accepting chemotaxis protein mcpA	TY90_06190	COG0840 Methyl-accepting chemotaxis protein
	Chemotaxis protein CheV	TY90_18185	COG0835 Chemotaxis signal transduction protein
	Methyl-accepting chemotaxis protein mcpB	TY90_00850	COG0840 Methyl-accepting chemotaxis protein
	Chemotaxis protein CheW	TY90_17610	COG0835 Chemotaxis signal transduction protein
	Chemotaxis protein methyltransferase	TY90_09925	COG1352 Methylase of chemotaxis methyl-accepting proteins
	Chemotaxis response regulator protein-glutamate methylesterase	TY90_17620	COG2201 Chemotaxis response regulator containing a CheY-like receiver domain and a methylesterase domain
	Flagellar motor switch protein FliG	TY90_15135	COG1536 Flagellar motor switch protein
	Methyl-accepting chemotaxis protein tlpA	TY90_21795	COG0840 Methyl-accepting chemotaxis protein
	Swarming motility protein swrAB	TY90_02810	COG0265 Trypsin-like serine proteases, typically periplasmic, contain C-terminal PDZ domain
	Flagellar assembly factor FliW	TY90_02710	function unknown
	Flagellar motor switch phosphatase FliY	TY90_15195	COG1776 Chemotaxis protein CheC, inhibitor of MCP methylation
	Flagellar biosynthetic protein fliZ	TY90_15205	COG3190 Flagellar biogenesis protein
	Flagellar biosynthetic protein fliR	TY90_15220	COG1684 Flagellar biosynthesis pathway, component FliR
	Probable flagellar assembly protein fliH	TY90_15140	COG1317 Flagellar biosynthesis/type III secretory pathway protein
	Flagellar biosynthetic protein fliP	TY90_15210	COG1338 Flagellar biosynthesis pathway, component FliP
	Flagellar protein FliT	TY90_02740	na
	Flagellar FliJ protein	TY90_15150	COG2882 Flagellar biosynthesis chaperone
	Flagellar protein FliL	TY90_15185	COG1580 Flagellar basal body-associated protein
	Flagellar motor switch protein FliG	TY90_15135	COG1536 Flagellar motor switch protein
	Flagellar hook-basal body complex protein FliE	TY90_15125	COG1677 Flagellar hook-basal body protein
	Flagellar biosynthetic protein FliQ	TY90_15215	COG1987 Flagellar biosynthesis pathway, component FliQ
	Flagellar protein fliS	TY90_02735	COG1516 Flagellin-specific chaperone FliS
Plant wall degrading enzymes and plant colonization	Putative aminopeptidase ysdC	TY90_10480	COG1363 Cellulase M and related proteins
	Putative aminopeptidase yhfE	TY90_04995	COG1363 Cellulase M and related proteins
	Putative aminopeptidase ytoP	TY90_07450	COG1363 Cellulase M and related proteins
	Endoglucanase	TY90_03065	COG2730 Endoglucanase
	Endoglucanase B	TY90_17520	COG2730 Endoglucanase
	Reducing end xylose-releasing exo-oligoxylanase	TY90_12415 [C]	COG3405 Endoglucanase Y
	Beta-glucosidase	TY90_12740	COG1472 Beta-glucosidase-related glycosidases
	Trehalose-6-phosphate hydrolase	TY90_06865	COG0366 Glycosidases
	Uncharacterized lipoprotein ybbD	TY90_09030	COG1472 Beta-glucosidase-related glycosidases
	Oligo-1,6-glucosidase 1	TY90_14210	COG0366 Glycosidases
	Intracellular maltogenic amylase	TY90_14180	COG0366 Glycosidases
	Pullulanase	TY90_07415	COG1523 Type II secretory pathway, pullulanase PulA and related glycosidases
	Alpha-amylase	TY90_16895	COG0366 Glycosidases
	Arabinan endo-1,5-alpha-L-arabinosidase	TY90_10485	COG3507 Beta-xylosidase
	Putative beta-xylosidase	TY90_13465 [C]	COG3507 Beta-xylosidase
	Xylose isomerase	TY90_01590	COG2115 Xylose isomerase
	Multiple sugar-binding periplasmic receptor ChvE	TY90_13115	COG4213 ABC-type xylose transport system, periplasmic component
	Arabinan endo-1,5-alpha-L-arabinosidase	TY90_15525	COG3507 Beta-xylosidase
	Uncharacterized protein yxiA	TY90_08160	COG3507 Beta-xylosidase
	Ribose transport system permease protein rbsC	TY90_02405	COG1172 Ribose/xylose/arabinose/galactoside ABC-type transport systems, permease components
	Xylose transport system permease protein xylH	TY90_13125	COG4214 ABC-type xylose transport system, permease component
	D-xylose-binding periplasmic protein	TY90_09370	COG4213 ABC-type xylose transport system, periplasmic component
Iron nutrition and metabolism	Rhizobactin siderophore biosynthesis protein rhbE	TY90_18465	COG3486 Lysine/ornithine N-monooxygenase
	Rhizobactin siderophore biosynthesis protein rhbD	TY90_18470	COG1670 Acetyltransferases, including N-acetylases of ribosomal proteins
	Probable siderophore transport system permease protein yfiZ	TY90_07875	COG0609 ABC-type Fe3 + −siderophore transport system, permease component
	Probable siderophore transport system permease protein yfhA	TY90_07870	COG0609 ABC-type Fe3 + −siderophore transport system, permease component
	Rhizobactin siderophore biosynthesis protein rhbC	TY90_18475	na
	Rhizobactin siderophore biosynthesis protein rhbF	TY90_18460	na
	Probable siderophore-binding lipoprotein yfiY	TY90_07880	COG0614 ABC-type Fe3 + −hydroxamate transport system, periplasmic component
	Probable siderophore-binding lipoprotein yfiY	TY90_08825	COG0614 ABC-type Fe3 + −hydroxamate transport system, periplasmic component
	Ferrous iron transport protein B	TY90_15330	COG0370 Fe2+ transport system protein B
	Iron(3+)-hydroxamate import system permease protein fhuG	TY90_12850	COG0609 ABC-type Fe3 + −siderophore transport system, permease component
	Iron-uptake system permease protein feuC	TY90_02125	COG0609 ABC-type Fe3 + −siderophore transport system, permease component
	Iron(3+)-hydroxamate-binding protein yxeB	TY90_11500	COG0614 ABC-type Fe3 + −hydroxamate transport system, periplasmic component
	Iron(3+)-hydroxamate import ATP-binding protein FhuC	TY90_12855	COG1120 ABC-type cobalamin/Fe3 + −siderophores transport systems, ATPase components
	Iron(3+)-hydroxamate-binding protein yxeB	TY90_15350	COG0614 ABC-type Fe3 + −hydroxamate transport system, periplasmic component
	Iron(3+)-hydroxamate-binding protein fhuD	TY90_13845	COG0614 ABC-type Fe3 + −hydroxamate transport system, periplasmic component
	Iron-uptake system-binding protein	TY90_02115	COG0614 ABC-type Fe3 + −hydroxamate transport system, periplasmic component
Phosphate nutrition and metabolism	Phosphate-binding protein pstS	TY90_12210	COG0226 ABC-type phosphate transport system, periplasmic component
	Phosphate import ATP-binding protein PstB 1	TY90_12230	COG1117 ABC-type phosphate transport system, ATPase component
	Sulfate permease CysP	TY90_14565	COG0306 Phosphate/sulphate permeases
	Probable low-affinity inorganic phosphate transporter	TY90_10295	COG0306 Phosphate/sulphate permeases
	Probable ABC transporter permease protein yqgI	TY90_12220	COG0581 ABC-type phosphate transport system, permease component
	Phosphate import ATP-binding protein PstB 2	TY90_12225	COG1117 ABC-type phosphate transport system, ATPase component
	Probable ABC transporter permease protein yqgH	TY90_12215	COG0573 ABC-type phosphate transport system, permease component
	Uncharacterized protein yqeW	TY90_11930	COG1283 Na+/phosphate symporter
	UPF0111 protein ykaA	TY90_10290	COG1392 Phosphate transport regulator (distant homolog of PhoU)
	Alkaline phosphatase 3	TY90_20965	COG1785 Alkaline phosphatase
	Probable manganese-dependent inorganic pyrophosphatase	TY90_02230	COG1227 Inorganic pyrophosphatase/exopolyphosphatase
	Uncharacterized protein ypjD	TY90_17920	COG1694 Predicted pyrophosphatase
	MazG Nucleotide Pyrophosphohydrolase	TY90_20180	COG1694 Predicted pyrophosphatase
	Bifunctional oligoribonuclease and PAP phosphatase nrnA	TY90_16540	COG0618 Exopolyphosphatase-related proteins
	Uncharacterized protein YhcW	TY90_11780	COG0637 Predicted phosphatase/phosphohexomutase
	Putative beta-phosphoglucomutase	TY90_14215	COG0637 Predicted phosphatase/phosphohexomutase
	Uncharacterized protein yvdC	TY90_08560	COG1694 Predicted pyrophosphatase
	Pyrophosphatase ppaX	TY90_02900	COG0546 Predicted phosphatases
	3-phytase	TY90_13145	na
Nitrogen uptake and metabolism	NifU-like protein	TY90_07755	COG0822 NifU homolog involved in Fe-S cluster formation
	Putative ammonium transporter sll0108	TY90_15930 [C]	COG0004 Ammonia permease
	Assimilatory nitrate reductase electron transfer subunit	TY90_04420	COG1251 NAD(P)H-nitrite reductase
	Nitrite reductase [NAD(P)H]	TY90_04410	COG1251 NAD(P)H-nitrite reductase
	Nitrate transporter	TY90_20435	COG2223 Nitrate/nitrite transporter
	Assimilatory nitrite reductase [NAD(P)H] small subunit	TY90_04405	COG2146 Ferredoxin subunits of nitrite reductase and ring-hydroxylating dioxygenases
	Uncharacterized transporter ywcJ	TY90_01155	COG2116 Formate/nitrite family of transporters
	Uncharacterized transporter yrhG	TY90_11795	COG2116 Formate/nitrite family of transporters
	Nitrate reductase beta chain	TY90_03135	COG1140 Nitrate reductase beta subunit
	Nitrate reductase gamma chain	TY90_03145	COG2181 Nitrate reductase gamma subunit
	Probable nitrate reductase molybdenum cofactor assembly chaperone NarJ	TY90_03140	COG2180 Nitrate reductase delta subunit
	Uncharacterized ABC transporter permease protein ytlD	TY90_07125	COG0600 ABC-type nitrate/sulfonate/bicarbonate transport system, permease component
	Nitrate transporter	TY90_20435	COG2223 Nitrate/nitrite transporter
	Putative aliphatic sulfonates transport permease protein ssuC	TY90_11560	COG0600 ABC-type nitrate/sulfonate/bicarbonate transport system, permease component
	Aliphatic sulfonates import ATP-binding protein SsuB	TY90_11550	COG1116 ABC-type nitrate/sulfonate/bicarbonate transport system, ATPase component
	Uncharacterized ABC transporter ATP-binding protein YtlC	TY90_07130	COG1116 ABC-type nitrate/sulfonate/bicarbonate transport system, ATPase component
	Putative aliphatic sulfonates-binding protein	TY90_1155	COG0715 ABC-type nitrate/sulfonate/bicarbonate transport systems, periplasmic components
Antibiotics and secondary metabolites production and transport, biocontrol-related genes	Surfactin synthase thioesterase subunit	TY90_12925	COG3208 Predicted thioesterase involved in non-ribosomal peptide biosynthesis
	Surfactin synthase subunit 2	TY90_21750	COG1020 Non-ribosomal peptide synthetase modules and related proteins
	Putative phosphoenolpyruvate synthase	TY90_00915	COG3319 Thioesterase domains of type I polyketide synthases or non-ribosomal peptide synthetases
	Surfactin synthase subunit 2	TY90_21285	COG1020 Non-ribosomal peptide synthetase modules and related proteins
	D-alanine--poly(phosphoribitol) ligase subunit 1	TY90_01430	COG1020 Non-ribosomal peptide synthetase modules and related proteins
	Putative phosphoenolpyruvate synthase	TY90_16790	COG3319 Thioesterase domains of type I polyketide synthases or non-ribosomal peptide synthetases
	Surfactin synthase subunit 1	TY90_17065	COG1020 Non-ribosomal peptide synthetase modules and related proteins
	Surfactin synthase subunit 3	TY90_12920	COG1020 Non-ribosomal peptide synthetase modules and related proteins
	Putative phosphoenolpyruvate synthase	TY90_00550	COG3319 Thioesterase domains of type I polyketide synthases or non-ribosomal peptide synthetases
	lantibiotic ABC transporter ATP-binding protein	TY90_19070	COG1131 ABC-type multidrug transport system, ATPase component
	lantibiotic ABC transporter ATP-binding protein	TY90_19920	COG1131 ABC-type multidrug transport system, ATPase component
	lantibiotic immunity protein	TY90_01250	na
	lantibiotic immunity protein	TY90_01260	na
	lantibiotic lichenicidin A1	TY90_01200	na
	lantibiotic lichenicidin A2	TY90_01205	na
	lantibiotic-modifying protein	TY90_01195	na
	lantibiotic-modifying protein	TY90_01210	na
	LanY	TY90_01235	na
	bacteriocin biosynthesis protein SagD	TY90_04705	COG1944 Uncharacterized conserved protein
	bacteriocin maturation protein	TY90_04710	na
	chitinase	TY90_20245	COG3979 Uncharacterized protein contain chitin-binding domain type 3
	chitinase	TY90_20250	COG3325 Chitinase
	Spermidine/putrescine transport system permease protein PotC	TY90_17055	COG1177 ABC-type spermidine/putrescine transport system, permease component II
	Spermidine/putrescine import ATP-binding protein PotA	TY90_17045	COG3839 ABC-type sugar transport systems, ATPase components
	Spermidine/putrescine-binding periplasmic protein 2	TY90_17040	COG0687 Spermidine/putrescine-binding periplasmic protein
	Spermidine synthase	TY90_01960	COG0421 Spermidine synthase
	acetoin dehydrogenase	TY90_13415	COG3284 Transcriptional activator \of acetoin/glycerol metabolism
	acetoin dehydrogenase	TY90_20060	na
	acetoin reductase	TY90_03165	COG1028 Dehydrogenases with different specificities (related to short-chain alcohol dehydrogenases)
	acetoin utilization protein AcuB	TY90_20055	COG0517 FOG: CBS domain
	acetoin:2,2 C6-dichlorophenolindophenol oxidoreductase subunit alpha	TY90_13395	COG1071 Pyruvate/2-oxoglutarate dehydrogenase complex, dehydrogenase (E1) component, eukaryotic type, alpha subunit
	acetolactate synthase	TY90_02385	COG0028 Thiamine pyrophosphate-requiring enzymes [acetolactate synthase, pyruvate dehydrogenase (cytochrome), glyoxylate carboligase, phosphonopyruvate decarboxylase]
	acetolactate synthase	TY90_05725	COG0440 Acetolactate synthase, small (regulatory) subunit
	acetolactate synthase catalytic subunit	TY90_05730	COG0440 Acetolactate synthase, small (regulatory) subunit
Oxidative stress response	Catalase X	TY90_01280	COG0753 Catalase
	Probable manganese catalase	TY90_03405	COG3546 Mn-containing catalase
	Glutathione peroxidase homolog BsaA	TY90_00120	COG0386 Glutathione peroxidase

## Discussion

Many microorganisms are reported as biocontrol agents acting as pathogen antagonists in the soil or inside plants. The mechanisms underlying this effect are not completely clear. Biocontrol bacteria act directly against pathogens producing many types of antimicrobial compounds, and indirectly on the plant host eliciting its protective response. In the discovery process of new bioactive strains, we focused on bacterial endophytes for their ability to spread along plants and colonize their inner tissues contrasting pathogens directly inside them.

In this work, we analyzed some traits of *Bacillus licheniformis* GL174 to assess its potential biocontrol activity. This bacterium, isolated from surface-sterilized leaves of *Vitis vinifera* cv. Glera, sampled in the Prosecco wine-making area, has been recognized as a *Bacillus licheniformis* strain [19]*.* Bacteria of the genus *Bacillus* are very common endophytes of a great variety of plant species [27, 28]. We then demonstrated that *Bacillus licheniformis* GL174 is a Glera endophyte - we assessed its ability to colonize Glera cuttings by using a GFP-tagged strain, following Koch’s postulate in the discovery of new endophytes. To this end, the ability of *Bacillus licheniformis* GL174 to colonize and inhabit stem tissues of Glera cuttings was evaluated 30 days post-inoculation. Successful visualization of *B. licheniformis* GL174 in the inner tissues showed that this strain is a true bacterial endophyte of *Vitis vinifera* cultivar Glera. According to Compant et al. [4], the colonization is not homogenous: bacteria are more often located in roots and stems rather than in leaves and reproductive structures where they tend to be more diluted. To check if *B. licheniformis* GL174 could be used as an endophytic biocontrol agent, we focused on its biochemical activities and genomic characteristics. Many endophytes can reduce infection by bacterial and fungal pathogens [5, 8, 12]. To potentially use this strain in grapevine cultivation, we checked if *B. licheniformis* GL174 could act against some fungal pathogens that severely damage vineyards in the Glera cultivation area. We tested in vitro the effect of the endophyte co-culturing *Bacillus licheniformis* GL174 and the specific grapevine fungal pathogens *Phaeoacremonium aleophilum, Botryosphaeria spp.* that are involved in the “*esca*” disease and other trunk diseases of grapevine plants [29, 30]*,* like *Botrytis cinerea,* which causes gray mold on grape, and against two general pathogens *Phytophthora infestans* and *Sclerotinia sclerotiorum*. In vitro tests demonstrated that the analyzed strain caused great mycelium growth reduction in *Botrytis cinerea*, *Phaeoacremonium aleophilum* and *Sclerotinia sclerotiorum*. Minor reduction was recorded in *Phytophtora infestans*. The inhibition percentages found in this work are comparable with other reduction effects reported for *Bacillus subtilis* and *Bacillus amyloliquefaciens* [31, 32]. To confirm this antifungal trait the strain was used in an in vivo test involving grapevine plants and the fungal pathogen *Botrytis cinerea*. The first part of the experiment, on the detached leaves, has demonstrated that the strain is effective against the pathogen when the leaves were infiltrated directly with the bacteria suspension. The antifungal effect of the bacteria could be due to nutritional competition and to the bacterial production of some diffusible molecules that can avoid or reduce pathogen growth [33]. Detached leaves from plants inoculated when cuttings (2 months before fungal infection) did not show any sign of protection. On the contrary, in the *in planta* experiment, both bacterial inoculation ways resulted effective for the plant protection. This indicates that the protective effect will probably be exerted by systemically induced defenses (i.e. by ISR), via activation of specific synthetic pathways that produce metabolites transferred through transport tissue protecting the plant from the gray mold. Detached infected leaves do not maintain the connection with the plant and thus leaves are not protected by the plant ISR, once infected by *Botrytis*. *Bacillus licheniformis* is reported in the literature as a producer of some lipopeptides [34] and chitinase [35], both of which may act as antifungal agents. The production of these molecules by *Bacillus licheniformis* GL174 was confirmed by tandem mass spectrometry. Spectra analysis showed that the endophyte constitutively produces many LP homologues of the lichenysin (5 compounds, Table 2) and surfactin (4 compounds, Table 2) families. Within each family there are many homologues that differ in chemical structure: linear and cyclic molecules, different acylic chain length, in amino acid in position 7, and/or methylation of some amino acid residues. Using these experimental conditions, no mycosubtilin production was detected when analyzing this strain. These results confirm that *Bacillus licheniformis* is a LP producer, as also demonstrated for a *B. licheniformis* strain isolated from marine sediments [36]. Moreover, this strain could be double effective for plant protection: in addition to the antimicrobial effects, molecules of the surfactin family are effectively recognized by plants eliciting an induced plant systemic response (ISR) that leads to increased pathogen tolerance [37–39]. Surfactins and lichenysins also have strong biosurfactant action, and help bacteria colonize and form biofilms as well as improve cell movements. On the other hand, this effect indirectly impairs colonization by other microorganisms such as pathogens [40]. Considering the results of the biochemical assays, we sequenced the whole strain genome, obtaining a large dataset of sequences to support previous results and provide further information about GL174 abilities. When analyzing coding sequence annotation, we identified a set of genes that complement the biochemical findings with genomic evidence, demonstrating the presence of some genes involved in plant-bacteria interaction and in plant pathogen biocontrol.

In analyzing the relationships between plant hosts and bacteria, we first evaluated the presence of genes involved in motility and chemotaxis, as recently suggested by Hardoim et al. [5]*.* The expression of these genes could lead to efficient plant colonization by bacteria. Moreover, with regard to plant colonization, we demonstrated the presence of cell-wall lytic enzymes. These enzymes can loosen the cell wall and help bacteria enter the apoplastic space: this bacterial ability could help efficient colonization by the GL174 strain and consequently exert its biocontrol activity directly from the inner tissues of plants [5, 41]. The presence of many genes for siderophore production and transport is another ability that can contribute to the biocontrolling effect of the strain. Indeed, these genes suggest that strain GL174 could efficiently compete with other microorganisms for iron nutrition, controlling the number of possible pathogens in the plant as shown for other PGP strains [41, 42]. Even though not strictly related to pathogen biocontrol, the ability to mobilize poorly bioavailable phosphate is also a remarkable feature of the strain. The presence of the 3-phytase gene suggests that strains like this could be used as biofertilizers in organic phosphate-rich soil [43]. In accordance with the mass spectrometry analysis of the lipopeptides, we identified the coding sequences of the three sub-units of the mega-enzyme responsible for the biosynthesis of the surfactin family lipopeptides-surfactin synthetase. This showed that the strain has genes to produce lipopeptides and that its set is expressed even in in vitro condition. Genome sequences were recognized by the COG analysis and UNiprot comparison as encoding proteins involved in the production and mobilization of bacteriocins. On the contrary to lipopeptides, these molecules are ribosomally-synthesized antibiotics, with a great structural diversity [44]. The analysis revealed the presence of putative enzymes for the class of lantibiotics (biosynthesis, modification and transport, see Table 3). All these genes, in addition to the gene encoding a chitinase, provide the bacterium a set of molecular weapons for biocontrol of pathogens. The presence of such different mechanisms of action against other microrganisms could be an effective strategy of antagonism affecting the development of efficient resistance strategies in the target organisms. The biocontrol effect of the strain could be exerted by the induction in the host plant of a systemic resistance response [5]. The genome analysis has revealed that the strain has the genes for production and transport of acetoin and 2,3-butanediol. Both these volatile compounds have been recognized as elicitors of plant resistance [45, 46]. Interestingly, 2,3-butanediol is also able to promote plant defence and biomass growth [47]. The presence in the genome of the spermidine synthase is another biocontrol-related trait of the strain. In fact, spermidine has biocontrol effects due to its action against biofilm formation. Moreover, this polyamine influences plant-bacteria interaction: it is recognized by plants and it can promote plant growth-modulating cell expansion and modulate hormonal balance [48]. In endophyte colonization, the bacterial expression of oxidative stress-related genes seems important to cope with the oxygen-reactive species that plants produce in their tissues [5]. The genome of strain GL174 contains different coding sequences for enzymes devoted to scavenging the oxidative burst, such as five different catalases and one glutathione peroxidase. These enzymes could enable bacteria to live inside plant tissues without being damaged by the oxidative burst of the plant. All these genomic data suggest that the strain can potentially be employed as a biocontrol agent. As the strain is a natural endophyte of Glera, it may proficiently and safely be used on grapevine in the field. This was confirmed by the results we obtained inoculating Glera cuttings, which were completely healthy and well colonized 30 days post-bacterization. The strain is also non-pathogenic for *Arabidopsis thaliana*, as previously demonstrated [20].

## Conclusions

The results of this integrated approach suggest that *Bacillus licheniformis* GL174 could act in *Vitis vinifera* Glera as a biocontrol agent, given its ability to inhibit fungal pathogen growth and to reduce the severity of fungal infection in vitro. This study is part of a project that aims to investigate the microbial biodiversity in the Veneto Glera vineyards to develop new agricultural practices and products for environmentally-friendly grapevine cultivation. *Bacillus licheniformis* GL174 is a good candidate to be tested in open field conditions to verify biocontrol effects on *Vitis vinifera* cv. Glera as well as other economically important crops.

## Additional file

Additional file 1: Dual plate assay. In vitro effect of *Bacillus licheniformis* GL174 on the plant pathogens *Phytophtora infestans* (A, B), and *Sclerotinia sclerotiorum* (C, D). Reduction of mycelium growth due to bacterium action (B, D) compared with negative controls without bacteria (A, C). (TIFF 1906 kb)

## Abbreviations

CDS: Coding sequence
COG: Cluster of orthologs
ESI-MS: Electrospray ionization mass spectrometry
GFP: Green fluorescent protein
ISR: Induced systemic resistance
LP: Lipopeptide
LSCM: Laser Scanning Confocal Microscopy
MFT: Major facilitator transporter
MS: Murashige and Skoog
NA: Nutrient Agar
NB: Nutrient broth
PGP: Plant growth promoting
PTS: Phosphotransferase System Familiy
U-HPLC: Ultra-High Performance Liquid Chromatography
