# Supplementary figures and images for: Biocontrol traits of Bacillus licheniformis GL174, a culturable endophyte of Vitis vinifera cv. Glera
Source: BMC Microbiol. 2018 Oct 16;18:133. doi: 10.1186/s12866-018-1306-5 (PMC6192205; doi:10.1186/s12866-018-1306-5)

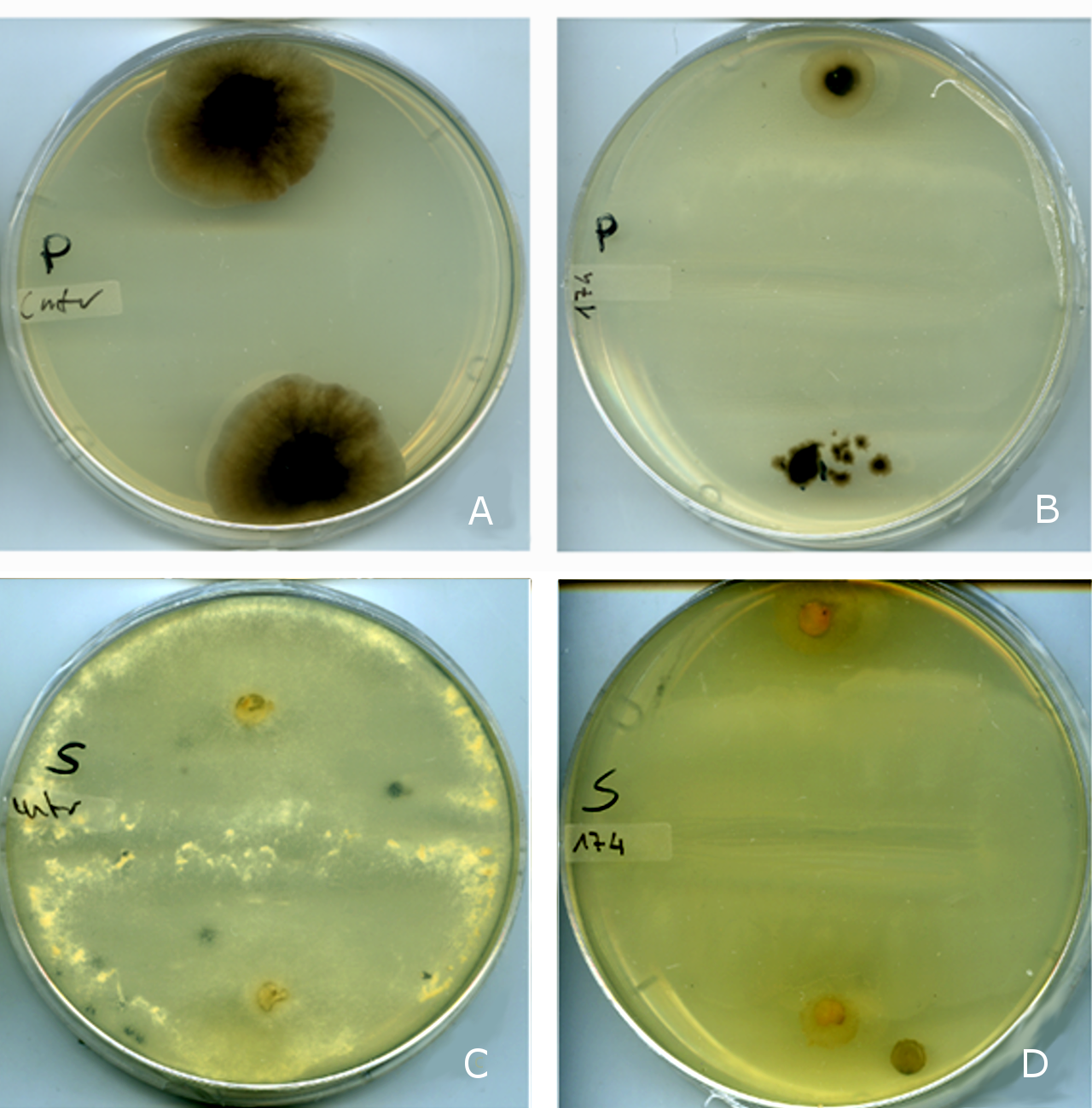

Supplement: Supplementary file 1 — Dual plate assay. In vitro effect of Bacillus licheniformis GL174 on the plant pathogens Phytophtora infestans (A, B), and Sclerotinia sclerotiorum (C, D). Reduction of mycelium growth due to bacterium action (B, D) compared with negative controls without bacteria (A, C). (TIFF 1906 kb) [file 12866_2018_1306_MOESM1_ESM.tiff]
